# Effectiveness of App-Delivered, Tailored Self-management Support for Adults With Lower Back Pain–Related Disability: A selfBACK Randomized Clinical Trial

**DOI:** 10.1001/jamainternmed.2021.4097

**Published:** 2021-08-02

**Authors:** Louise Fleng Sandal, Kerstin Bach, Cecilie K. Øverås, Malene Jagd Svendsen, Tina Dalager, Jesper Stejnicher Drongstrup Jensen, Atle Kongsvold, Anne Lovise Nordstoga, Ellen Marie Bardal, Ilya Ashikhmin, Karen Wood, Charlotte Diana Nørregaard Rasmussen, Mette Jensen Stochkendahl, Barbara I. Nicholl, Nirmalie Wiratunga, Kay Cooper, Jan Hartvigsen, Per Kjær, Gisela Sjøgaard, Tom I. L. Nilsen, Frances S. Mair, Karen Søgaard, Paul Jarle Mork

**Affiliations:** 1 Department of Sports Science and Clinical Biomechanics, University of Southern Denmark, Odense, Denmark; 2 Department of Computer Science, Norwegian University of Science and Technology, Trondheim, Norway; 3 Department of Public Health and Nursing, Norwegian University of Science and Technology, Trondheim, Norway; 4 Musculoskeletal Disorders and Physical Workload, National Research Centre for the Working Environment, Copenhagen, Denmark; 5 Institute of Health and Wellbeing, University of Glasgow, Glasgow, United Kingdom; 6 Nordic Institute of Chiropractic and Clinical Biomechanics, Odense, Denmark; 7 Robert Gordon University School of Computing, Aberdeen, United Kingdom; 8 Robert Gordon University School of Health Sciences, Aberdeen, United Kingdom

## Abstract

**Importance:**

Lower back pain (LBP) is a prevalent and challenging condition in primary care. The effectiveness of an individually tailored self-management support tool delivered via a smartphone app has not been rigorously tested.

**Objective:**

To investigate the effectiveness of selfBACK, an evidence-based, individually tailored self-management support system delivered through an app as an adjunct to usual care for adults with LBP-related disability.

**Design, Setting, and Participants:**

This randomized clinical trial with an intention-to-treat data analysis enrolled eligible individuals who sought care for LBP in a primary care or an outpatient spine clinic in Denmark and Norway from March 8 to December 14, 2019. Participants were 18 years or older, had nonspecific LBP, scored 6 points or higher on the Roland-Morris Disability Questionnaire (RMDQ), and had a smartphone and access to email.

**Interventions:**

The selfBACK app provided weekly recommendations for physical activity, strength and flexibility exercises, and daily educational messages. Self-management recommendations were tailored to participant characteristics and symptoms. Usual care included advice or treatment offered to participants by their clinician.

**Main Outcomes and Measures:**

Primary outcome was the mean difference in RMDQ scores between the intervention group and control group at 3 months. Secondary outcomes included average and worst LBP intensity levels in the preceding week as measured on the numerical rating scale, ability to cope as assessed with the Pain Self-Efficacy Questionnaire, fear-avoidance belief as assessed by the Fear-Avoidance Beliefs Questionnaire, cognitive and emotional representations of illness as assessed by the Brief Illness Perception Questionnaire, health-related quality of life as assessed by the EuroQol-5 Dimension questionnaire, physical activity level as assessed by the Saltin-Grimby Physical Activity Level Scale, and overall improvement as assessed by the Global Perceived Effect scale. Outcomes were measured at baseline, 6 weeks, 3 months, 6 months, and 9 months.

**Results:**

A total of 461 participants were included in the analysis; the population had a mean [SD] age of 47.5 [14.7] years and included 255 women (55%). Of these participants, 232 were randomized to the intervention group and 229 to the control group. By the 3-month follow-up, 399 participants (87%) had completed the trial. The adjusted mean difference in RMDQ score between the 2 groups at 3 months was 0.79 (95% CI, 0.06-1.51; *P* = .03), favoring the selfBACK intervention. The percentage of participants who reported a score improvement of at least 4 points on the RMDQ was 52% in the intervention group vs 39% in the control group (adjusted odds ratio, 1.76; 95% CI, 1.15-2.70; *P* = .01).

**Conclusions and Relevance:**

Among adults who sought care for LBP in a primary care or an outpatient spine clinic, those who used the selfBACK system as an adjunct to usual care had reduced pain-related disability at 3 months. The improvement in pain-related disability was small and of uncertain clinical significance. Process evaluation may provide insights into refining the selfBACK app to increase its effectiveness.

**Trial Registration:**

ClinicalTrials.gov Identifier: NCT03798288

## Key Points

**Question:**

Is selfBACK, an evidence-based, individually tailored self-management support system that is delivered through an artificial intelligence–based app and in conjunction with usual care, effective for pain-related disability in adults with lower back pain?

**Findings:**

In this randomized clinical trial involving 461 participants in Denmark and Norway, those who received the selfBACK intervention had reduced pain-related disability compared with those who received usual care alone. However, the effect may be too small to be clinically meaningful.

**Meaning:**

The findings of this trial and process evaluation may inform and encourage further development of the selfBACK intervention to increase its effectiveness.

## Teaser

This randomized clinical trial examines a decision support tool that provides lower back pain information and self-management recommendations that are specific to an individual’s characteristics, symptoms, and symptom progression.

## Introduction

Lower back pain (LBP) is the leading cause of disability worldwide, and its burden is expected to grow in the coming decades.^1,2,3^ In the United States, LBP accounts for at least 264 million lost workdays per year, equating to more than 2 lost workdays per year for every full-time employee.^4^ Despite the vast amount of allocated health care resources, the burden of LBP has increased substantially over the past 3 decades.^5^ In 2016, lower back and neck pain accounted for the highest amount of health care spending in the United States.^6^

In the United States, LBP is the third most common reason for individuals to visit their primary care physician.^7^ A specific cause of LBP can rarely be identified and is most often diagnosed as being nonspecific.^8^ Evidence-based self-management support that is tailored to the needs and abilities of the patient is recommended as part of the first-line treatment for nonspecific LBP.^9,10,11,12^ This support includes providing patients with adequate information, reassurance, and education as well as advice to maintain daily activities and exercise regularly.^11,13^ However, primary care physicians generally lack the time, resources, and training to deliver such support,^14^ and adherence to self-management recommendations without feedback or reinforcement is challenging for most patients.^15^ Smartphone technology along with knowledge-driven artificial intelligence (AI) can be used to make tailored self-management support available to patients.^16^ A recent meta-analysis of randomized clinical trials concluded that e-health programs may be beneficial in LBP self-management.^17^

Informed by current best clinical evidence and knowledge-driven AI, we developed selfBACK, an innovative decision support system, to facilitate, improve, and reinforce self-management of LBP.^18^ In this randomized clinical trial, we investigated the effectiveness of selfBACK, an evidence-based, individually tailored self-management support system delivered via an app as an adjunct to usual care for adults with LBP-related disability who sought care in a primary care or an outpatient spine clinic. We hypothesized that patients who were randomized to receive the selfBACK intervention would have a lower LBP-related disability score and favorable other outcomes after 3 months compared with those who were randomized to receive usual care alone.

## Methods

### Study Design, Setting, and Participants 

The randomized clinical trial was approved by the Danish Data Protection Agency and regional ethics committees in Denmark and Norway. All potential participants provided written informed consent before trial enrollment. The trial protocol has been published elsewhere.^19,20^ We followed the Consolidated Standards of Reporting Trials (CONSORT) reporting guideline.

We recruited adults who were 18 years or older, had nonspecific LBP within the preceding 8 weeks, scored 6 points or higher on the Roland-Morris Disability Questionnaire (RMDQ) at the time of screening, had consulted a clinician (general practitioner, physiotherapist, or chiropractor) in the region of Southern Denmark or in the Trondheim municipality in Norway or had undergone a clinical examination at an outpatient spine clinic (Spine Centre of Southern Denmark), had a smartphone (with an iOS or Android operating system), and had access to email. Exclusion criteria were the inability to carry out the intervention (ie, problems with speaking, reading, or understanding Danish or Norwegian; mental or physical conditions that limited participation; or inability to perform physical exercise), fibromyalgia, previous spinal surgery, current pregnancy, current participation in other LBP-focused research, or an RMDQ score lower than 6 points at screening.

**Figure 1.**
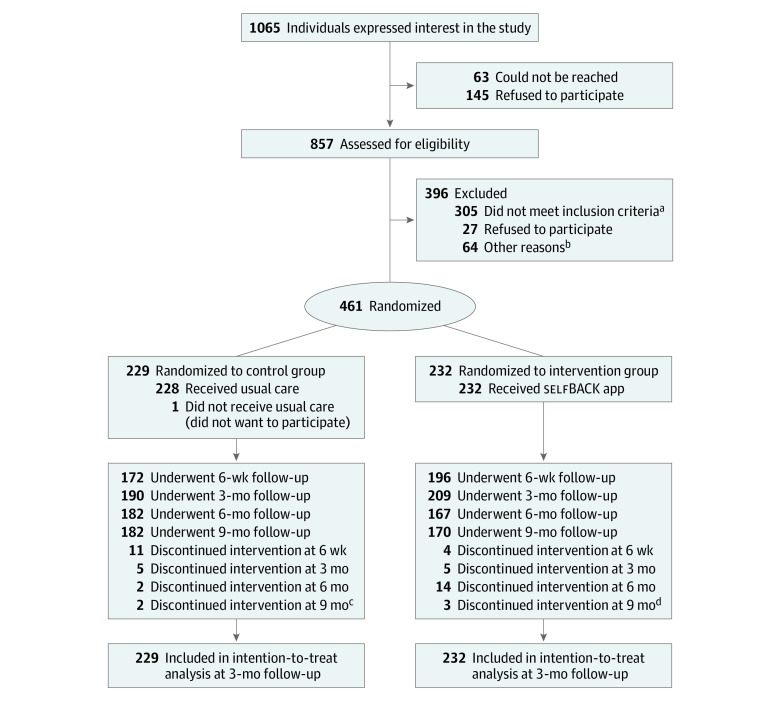
CONSORT Diagram ^a^Individuals were excluded for having no lower back pain (n = 48), a Roland-Morris Disability Questionnaire score lower than 6 points (n = 235), and inadequate smartphone (n = 22). ^b^Other reasons for exclusion were being younger than 18 years (n = 2); being unable to speak, read, or understand the national language (n = 2); having mental or physical conditions that limited participation (n = 12); being unable to take part in exercise or physical activity (n = 5); having fibromyalgia diagnosis (n = 11); participating currently in other lower back research (n = 2); and having previous back surgery (n = 30). ^c^Reasons for discontinuation of usual care included work pressure (n = 2), randomization to usual care (n = 2), unknown reasons (n = 10), personal reasons (n = 1), lack of time (n = 2), questionnaire issues (n = 1), not understanding the concept (n = 1), and “too much hassle” (n = 1). ^d^Reasons for discontinuation of the selfBACK intervention included work pressure (n = 2), knee injury (n = 1), unknown reasons (n = 12), surgery (n = 2), personal reasons (n = 1), lack of time (n = 1), questionnaire issues (n = 2), technical issues with app or wristband (n = 4), and starting other new treatment (n = 1).

Eligible individuals were enrolled between March 8 to December 14, 2019. Figure 1 shows the flow of participants through the trial.

### Randomization to Intervention Group or Control Group

Participants completed a web-based questionnaire and were thereafter randomized. A web-based trial management system, administered by the Unit for Applied Clinical Research, Faculty of Medicine and Health Sciences of the Norwegian University of Science and Technology, was used in randomization. Group allocation was concealed by the trial management system until the randomization was performed. Participants were randomized in a 1:1 ratio using permuted blocks with random sizes from 4 to 20 and stratified by country (Denmark or Norway) and clinician (general practitioner, physiotherapist, chiropractor, or outpatient clinic). Participants were not blinded to group allocation after randomization. Participants who were randomized to receive usual care (control group) were instructed to manage their LBP according to the advice or treatment offered by their clinician. Participants who were randomized to receive the selfBACK self-management support system in addition to usual care (intervention group) were instructed to install the AI-based selfBACK app to their smartphone and to wear a step-detecting wristband (Mi Band 3; Xiaomi) that was connected to the app. The app also works with other commercially available step-detecting wristbands and the built-in step counter in smartphones. A research assistant (including some of us: L.F.S., C.K.Ø., T.D., J.S.D.J., A.K., A.L.N., and E.M.B.) guided the app installation and briefly introduced the app functions in a face-to-face meeting with the participant. Participants were instructed to use the app at their convenience and for as long as they needed, and no directives were given regarding the end point of use. They were informed that the app was a supplement to their usual care and that they should follow any advice given by their clinician.

The selfBACK intervention is an evidence-based decision support system that provides weekly, individually tailored self-management recommendations for 3 main components that are endorsed by clinical guidelines^11,12,21^: (1) physical activity (number of steps), (2) strength and flexibility exercises, and (3) daily educational messages. In addition, the app provides general information about LBP and access to several tools (goal setting, mindfulness audios, pain-relieving exercises, and sleep reminders) that participants could use at their convenience.

A detailed description of the intervention is presented in the eAppendix in Supplement 1. The development of the evidence-based content as well as the design, architecture, and functions of the selfBACK system have been described in detail elsewhere.^18,22^ Briefly, the weekly self-management recommendations are tailored to the participant’s characteristics, symptoms, and symptom progression, which are reported through the app by using case-based reasoning,^23^ a branch of knowledge-driven AI.^24,25^ In the selfBACK system, the core of case-based reasoning is knowledge of previous successful cases along with data about the current case, which enables the system to provide patient-centered recommendations based on current needs and past interventions that proved effective. By following the weekly recommendations, participants could collect badges and rewards that are displayed on the app. In this trial, encouraging and commending push notifications, triggered by the participants’ behaviors, were sent to the participants’ smartphones to motivate and reinforce the desired behavior.

### Outcomes and Follow-up

Outcomes were evaluated using a web-based questionnaire at baseline, 6 weeks, 3 months, 6 months, and 9 months. Sociodemographic information was collected at baseline (Table 1). Following the prespecified statistical analysis plan (Supplement 2), we assessed the primary outcome as the mean difference in RMDQ scores between the intervention group and control group at 3 months. The RMDQ is a reliable and valid measure of pain-related disability in people with nonspecific LBP.^26^ The RMDQ scale ranges from 0 to 24 points, with higher scores indicating more pain-related disability. In addition, we examined the difference in the percentage of participants who reported achieving at least a 2- or 4-point improvement in RMDQ score. There is no clear consensus on what constitutes a clinically meaningful change on the RMDQ scale, but several studies have indicated that meaningful change is likely to be a score ranging from 2 to 4 points.^27,28,29,30^

**Table 1.** Baseline Characteristics of the Study Participants

Variable	No. (%)		
	All participants (N = 461)	Control group: usual care (n = 229)	Intervention group: selfBACK system (n = 232)
Sociodemographic characteristics			
Age, mean (SD) [range], y	47.5 (14.7) [18-86]	46.7 (14.4) [18-81]	48.3 (15.0) [20-86]
BMI, mean (SD) [range]	27.6 (5.1) [17-54]	27.8 (5.4) [18-54]	27.3 (4.7) [17-46]
Female sex	255 (55)	134 (59)	121 (52)
Male sex	206 (45)	95 (41)	111 (48)
Educational achievement: >12 y	297 (64)	145 (63)	152 (66)
Full-time employment	281 (61)	143 (62)	138 (59)
Married or living with partner	332 (72)	158 (69)	174 (75)
Clinical setting of patient recruitment			
General practitioner	68 (15)	34 (15)	34 (15)
Physiotherapist	135 (29)	67 (29)	68 (29)
Chiropractor	160 (35)	79 (35)	81 (35)
Outpatient back clinic	98 (21)	49 (21)	49 (21)
LBP history			
Duration of current pain episode: >12 wk	267 (58)	136 (59)	131 (56)
No. of days with LBP in past year			
1-7	17 (4)	6 (3)	11 (5)
8-30	61 (13)	33 (14)	28 (12)
>30	186 (40)	90 (39)	96 (41)
Daily	197 (43)	100 (44)	97 (42)
Use of pain medication			
None	94 (20)	50 (22)	44 (19)
1-2 d	85 (18)	39 (17)	46 (20)
3-5 d	125 (27)	66 (29)	59 (25)
Daily	157 (34)	74 (32)	83 (36)
Baseline measure of primary outcome			
RMDQ score, range: 0-24, mean (SD)	10.4 (4.4)	10.6 (4.4)	10.3 (4.4)
Baseline measures of secondary outcomes			
LBP intensity level, NRS range: 0-10, mean (SD)			
Average pain intensity level in past week	4.9 (1.9)	4.9 (1.9)	4.8 (2.0)
Worst pain intensity level in past week	6.6 (1.9)	6.6 (2.0)	6.6 (1.9)
PSEQ score, range: 0-60, mean (SD)	44.1 (11.1)	43.6 (11.2)	44.6 (10.9)
FABQ score, range: 0-24, mean (SD)	10.3 (5.4)	10.2 (5.2)	10.5 (5.7)
BIPQ score, range: 0-80, mean (SD)	44.0 (10.9)	45.3 (10.4)	42.8 (11.2)
EQ-VAS score, range: 0-100, mean (SD)	66.2 (16.5)	65.2 (16.7)	67.1 (16.3)
EQ-5D weighted score, range: −0.62 to 1.00, mean (SD)	0.70 (0.13)	0.70 (0.14)	0.71 (0.11)
SGPALS			
Sedentary	33 (7)	18 (8)	15 (6)
Some physical activity	239 (52)	121 (53)	118 (51)
Global Perceived Effect scale score, range: −5 to 5	NA	NA	NA

Abbreviations: BIPQ, Brief Illness Perception Questionnaire; BMI, body mass index (calculated as weight in kilograms divided by height in meters squared); EQ-5D, EuroQol-5 Dimension; EQ-VAS, EuroQol visual analog scale; FABQ, Fear-Avoidance Beliefs Questionnaire; LBP, lower back pain; NA, not applicable; NRS, numerical rating scale; PSEQ, Pain Self-Efficacy Questionnaire; RMDQ, Roland-Morris Disability Questionnaire; SGPALS, Saltin-Grimby Physical Activity Level Scale.

Prespecified secondary outcomes included average and worst LBP intensity levels in the preceding week as measured on the numerical rating scale (range: 0-10, with higher scores indicating higher intensity)^31^; confidence in ability to cope despite pain as assessed with the Pain Self-Efficacy Questionnaire (range: 0-60, with higher scores indicating greater confidence)^32^; fear-avoidance belief as assessed by the Fear-Avoidance Beliefs Questionnaire physical activity subscale (range: 0-24, with higher scores indicating greater fear)^33^; cognitive and emotional representations of illness as assessed by the Brief Illness Perception Questionnaire (range: 0-80, with higher scores indicating greater illness perception)^34^; health-related quality of life as assessed by the EuroQol-5 Dimension questionnaire, weighted according to the Danish value set (range: −0.62 to 1.00, with higher scores indicating better health status),^35^ and the EuroQol visual analog scale (range: 0-100, with higher scores indicating better health status)^36^; leisure time physical activity level as assessed by the Saltin-Grimby Physical Activity Level Scale (4 categories: sedentary, some physical activity, regular physical activity, and regular hard physical activity)^37^; and overall improvement as assessed by the Global Perceived Effect scale (range: −5 to 5, with scores above 0 points indicating improvement [anchor: “very much better”] and scores below 0 points indicating worsening [anchor: “very much worse”]).^38^ In line with the prespecified statistical analysis plan, we examined a set of additional secondary and exploratory outcomes.

### Adverse Events and Power

Participant-reported occurrences of harms and adverse events were registered and discussed in weekly trial management meetings.

The planned sample size of at least 350 participants (175 in each group) was based on a power of 90% to detect a 2-point mean group difference in RMDQ score at 3 months, assuming an SD of 6 points, a correlation of 0.4 between repeated measures in the same participants, a 2-sided α = .05, and a 30% dropout rate during follow-up.^19^

### Statistical Analysis 

The primary intention-to-treat analysis estimated the mean group difference in RMDQ score using a constrained longitudinal data analysis,^39,40^ in which both the baseline and all follow-up values were modeled as dependent variables. The baseline means were constrained to be equal for both groups, which was reasonable because of the randomization, and the analyses were thus adjusted for any baseline difference in the outcome variable. The model included a random intercept for each participant to account for the dependency in observations within participants over time. Results were presented as mean differences with 95% CIs between the intervention group and control group at 3- and 9-month follow-up. Following evidence-based recommendations,^41,42^ we adjusted all effect estimates for variables used to stratify the randomization (by country and clinician) and for potentially important predictors of the outcome (age [years], sex [male vs female], educational achievement [<10, 10-12, or >12 years], duration of current pain episode [<1, 1-4, 5-12, or >12 weeks], and average pain intensity level in the past week at baseline [0-10 scale]).

Preplanned sensitivity analyses of the primary outcome included (1) multiple imputations of missing values using a multivariate normal approach and 20 imputed data sets; (2) a complete case analysis, including participants with data at all time points; and (3) a per protocol analysis, including adherent participants in the intervention group (adherence was defined as creating ≥6 self-management plans during the first 12 weeks after randomization). We assessed the assumptions related to the normality and homogeneity of residuals as well as the normality of random intercepts for all models. Analysis of mean group differences in secondary outcomes followed the same analytic approach.

We used a generalized estimated equation logistic model to estimate the odds ratio (OR) for achieving at least a 2- or 4-point improvement in RMDQ score from baseline to each follow-up time point. Similar analyses were performed to estimate the ORs for secondary binary outcomes that were classified according to clinically meaningful cutoffs. The number needed to treat was calculated as the inverted risk difference from a generalized estimated equation Poisson model. For all generalized estimated equation models, an exchangeable correlation structure was assumed and a robust variance estimator was used.

All estimates of precision were based on 2-sided tests. Statistical significance was defined as a 2-sided *P* < .05. All analyses were performed using Stata, version 16.1 (StataCorp LLC).

## Results

Among the 1065 individuals who expressed interest in the study, 461 were randomized to the intervention group (n = 232) or the control group (n = 229) (Figure 1). Overall, 317 participants (69%) were recruited in Denmark and 144 participants (31%) were recruited in Norway. The primary reason for exclusion was an RMDQ score lower than 6 points (n = 235). Among participants in the intervention group, 181 (78%) adhered to selfBACK in addition to the usual care intervention. Complete data on the RMDQ were obtained from 368 participants (80%) at 6 weeks, 399 (87%) at 3 months, 349 (76%) at 6 months, and 352 (76%) at 9 months.

Among study participants, 255 (55%) were women, 206 (45%) were men, the mean (SD) age was 47.5 (14.7) years, and the mean (SD) body mass index (calculated as weight in kilograms divided by height in meters squared) was 27.6 (5.1) (Table 1). Sociodemographic characteristics, LBP history, and primary and secondary outcome scores were similar between the 2 groups at baseline (Table 1). None of the participants reported any harms or adverse events.

### Primary Outcome

**Figure 2.**
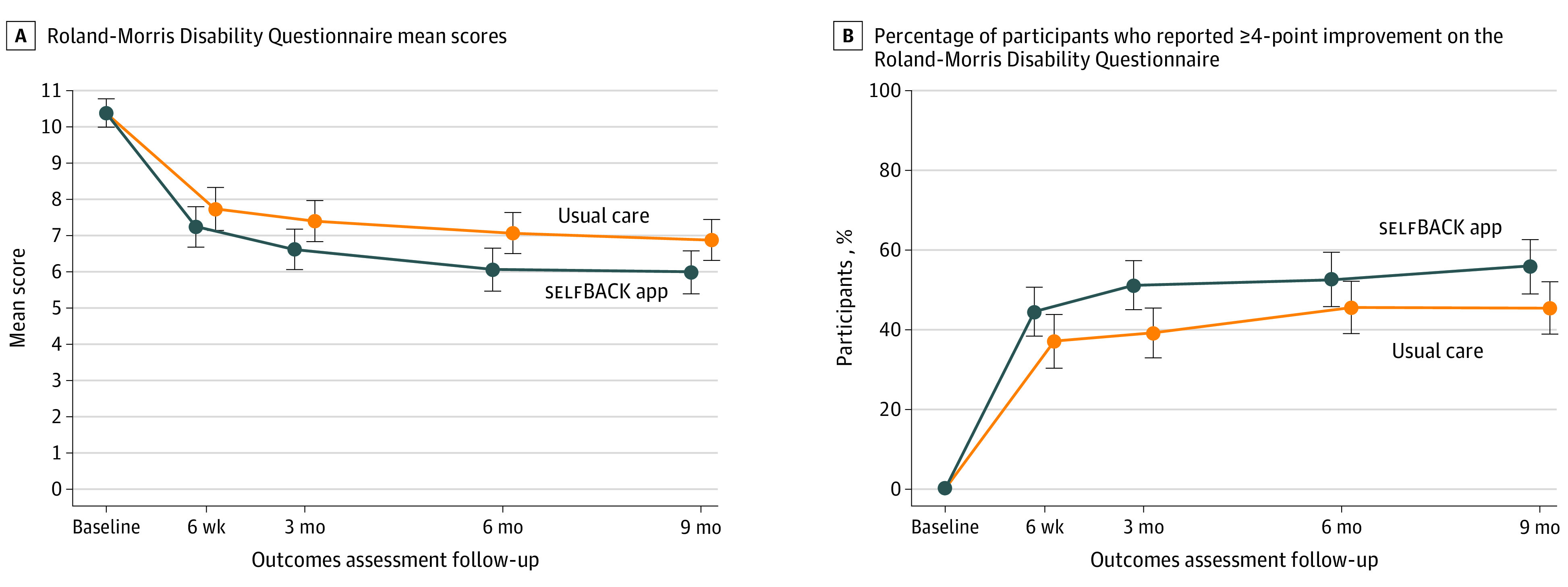
Roland-Morris Disability Questionnaire Scores and Reported Score Improvement at All Time Points Error bars represent 95% CIs.

From baseline to 3 months, the within-group mean (SD) change in RMDQ score was 3.0 (4.5) points for the control group and 3.7 (4.5) points for the intervention group. At 3 months, the adjusted mean RMDQ score was −0.79 (95% CI, −1.51 to −0.06; *P* = .03) points lower in the intervention group compared with the control group (Table 2). This effect was sustained at 9 months (score, −0.88; 95% CI, −1.64 to −0.11 points) (Table 2, Figure 2, and eTable 1 in Supplement 1) but was somewhat attenuated in sensitivity analyses (score, −0.78; 95% CI, −1.54 to −0.03 points) (eTable 1 in Supplement 1).

**Table 2.** Differences in Primary and Secondary Outcomes at 3- and 9-Month Follow-up

Variable	Mean (SD)^a^			Between-group differences, adjusted mean score (95% CI)^c^
	All participants (N = 461)	Control group: usual care (n = 229)	Intervention group: selfBACK system (n = 232)^b^	
**Primary outcome**				
RMDQ score				
Baseline	10.4 (4.4)			NA
3-mo Follow-up		7.4 (5.4)	6.7 (4.7)	−0.79 (−1.51 to −0.06)
9-mo Follow-up		6.9 (5.6)	6.0 (5.3)	−0.88 (−1.64 to −0.11)
**Secondary outcomes**				
Average pain intensity level in preceding wk, score range: 0-10				
Baseline	4.9 (1.9)			NA
3-mo Follow-up		3.9 (2.4)	3.3 (2.2)	−0.62 (−0.99 to −0.26)
9-mo Follow-up		3.7 (2.4)	3.0 (2.3)	−0.69 (−1.07 to −0.30)
Worst pain intensity level in preceding wk, score range: 0-10				
Baseline	6.6 (1.9)			NA
3-mo Follow-up		5.2 (2.7)	4.4 (2.5)	−0.73 (−1.15 to −0.31)
9-mo Follow-up		5.0 (2.8)	4.0 (2.6)	−1.00 (−1.45 to −0.56)
PSEQ score, range: 0-60				
Baseline	44.1 (11.0)			NA
3-mo Follow-up		46.6 (11.2)	49.2 (9.9)	2.52 (1.04 to 3.99)
9-mo Follow-up		46.9 (11.0)	50.2 (9.7)	3.25 (1.71 to 4.79)
FABQ score, range: 0-24				
Baseline	10.3 (5.4)			NA
3-mo Follow-up		9.1 (5.4)	8.6 (5.6)	−0.43 (−1.34 to 0.48)
9-mo Follow-up		8.7 (5.6)	7.8 (5.5)	−0.83 (−1.79 to 0.13)
BIPQ score, range: 0-80				
Baseline	44.0 (10.9)			NA
3-mo Follow-up		40.4 (13.5)	35.8 (14.2)	−4.57 (−6.42 to −2.72)
9-mo Follow-up		38.0 (14.9)	34.1 (14.9)	−3.88 (−5.81 to −1.95)
EQ-VAS score, range: 0-100				
Baseline	66.2 (16.5)			NA
3-mo Follow-up		70.6 (17.4)	70.9 (16.9)	0.36 (−2.42 to 3.14)
9-mo Follow-up		71.9 (17.9)	73.4 (16.1)	1.54 (−1.38 to 4.45)
EQ-5D weighted score, range: −0.6 to 1.0				
Baseline	0.70 (0.13)			NA
3-mo Follow-up		0.74 (0.13)	0.76 (0.12)	0.02 (−0.01 to 0.04)
9-mo Follow-up		0.76 (0.14)	0.78 (0.13)	0.02 (0.00 to 0.05)
Global Perceived Effect scale score, range: −5 to 5				
Baseline	NA			NA
3-mo Follow-up		1.2 (1.9)	2.0 (1.9)	0.70 (0.39 to 1.01)
9-mo Follow-up		1.3 (2.2)	2.2 (2.0)	0.81 (0.49 to 1.15)

Abbreviations: BIPQ, Brief Illness Perception Questionnaire; EQ-5D, EuroQol-5 Dimension; EQ-VAS, EuroQol visual analog scale; FABQ, Fear-Avoidance Beliefs Questionnaire; NA, not applicable; PSEQ, Pain Self-Efficacy Questionnaire; RMDQ, Roland-Morris Disability Questionnaire.

^a^ Marginal means were from a crude linear mixed model, and SDs were from raw data among persons with information at the specific time points.

^b^ App-delivered self-management support in addition to usual care.

^c^ Adjusted for stratification variables (country and clinician), educational achievement (<10, 10-12, or >12 years), duration of current pain episode (<1, 1-4, 5-12, or >12 weeks), average pain intensity level in the past week at baseline (continuous, range: 0-10), sex (male vs female), and age (years).

The percentage of participants who reported a score improvement of at least 4 points on the RMDQ from baseline to 3 months was 52% (n = 108 of 209 participants) in the intervention group vs 39% (n = 74 of 190 participants) in the control group, corresponding to an adjusted OR for improvement in the intervention group of 1.76 (95% CI, 1.15-2.70; *P* = .01) (Table 3 and Figure 2) compared with the control group. This result corresponded to a number needed to treat of 7.3 (95% CI, 4.3-24.1). Analysis for the score improvement of 2 points or more is presented in eTable 2 in Supplement 1.

**Table 3.** Proportion of Participants Who Reported Improvement and Group Comparisons at 3- and 9-Month Follow-up

Reported ≥4-point improvement on RMDQ	Control group: usual care		Intervention group: selfBACK system^a^		Between-group differences, OR (95% CI)^b^^,^^c^
	No. of participants reporting improvement/No. of participants (% reporting)	OR (95% CI)^b^	No. of participants reporting improvement/No. of participants (% reporting)	OR (95% CI)^b^	
Baseline	0/229 (NA)	NA	0/232 (NA)	NA	NA
3-mo follow-up	74/190 (39)	1.11 (0.77- 1.61)	108/209 (52)	1.96 (1.25-3.07)	1.76 (1.15-2.70)
9-mo follow-up	82/182 (45)	1.50 (1.05-2.14)	95/170 (56)	2.45 (1.53-3.92)	1.63 (1.04-2.55)

Abbreviations: NA, not applicable; OR, odds ratio; RMDQ, Roland-Morris Disability Questionnaire.

^a^ App-delivered self-management support in addition to usual care.

^b^ Adjusted for stratification variables (country and clinician), educational achievement (<10, 10-12, or >12 years), duration of current pain episode (<1, 1-4, 5-12, or >12 weeks), average pain intensity level in the past week at baseline (continuous, range: 0-10), sex (male vs female), and age (years).

^c^ Usual care was used as reference group.

### Secondary Outcomes

At 3 months, between-group differences in favor of the intervention group were observed for average pain intensity level in the preceding week (−0.62; 95% CI, −0.99 to −0.26; *P* = .001), worst pain intensity level in the preceding week (−0.73; 95% CI, −1.15 to −0.31; *P* = .001), Pain Self-Efficacy Questionnaire score (2.52; 95% CI, 1.04-3.99; *P* = .001), Brief Illness Perception Questionnaire score (−4.57; 95% CI, −6.42 to −2.72; *P* < .001), and Global Perceived Effect scale score (0.70; 95% CI, 0.39-1.01; *P* < .001) (Table 2). Fear-avoidance beliefs, health-related quality of life (Table 2), and physical activity (eTable 3 in Supplement 1) did not differ between groups at 3 months. The between-group differences for the secondary outcomes were sustained at 9 months (Table 2 and eTable 3 in Supplement 1), although the differences were smaller than previously reported as clinically relevant for populations with LBP. Exploratory outcomes are reported in eTables 4 and 5 in Supplement 1.

## Discussion

Among adults who sought care for LBP, those who were randomized to receive selfBACK, an evidence-based and individually tailored self-management support system delivered through an AI-based app as an adjunct to usual care, showed reduced pain-related disability at 3 months compared with those who were randomized to receive usual care alone. However, this effect was less than the expected 2-point score improvement on the RMDQ. The clinical significance of this finding is therefore uncertain, although a larger percentage of participants in the intervention group achieved a clinically meaningful score improvement of 4 points or higher on the RMDQ at 3 months compared with the control group (52% vs 39%). Between-group differences for the secondary outcomes at 3 months favored the intervention, but the effects were small. Overall, the results for the primary and secondary outcomes were sustained at 9 months.

To our knowledge, this randomized clinical trial was the first to use an AI-based app to deliver evidence-based and individually tailored self-management support to adults with LBP. Previously, AI was used in LBP classification but not for prognosis or guiding treatment.^43^ The results of the current trial complement evidence from previous systematic reviews of randomized clinical trials that showed that nonpharmacological active treatments, such as exercise or mindfulness-based stress reduction, may ease LBP-related disability.^44^ Furthermore, a recent meta-analysis concluded that digital support systems may be beneficial in LBP self-management.^17^

Although no general consensus on this issue has been reached, a clinically relevant score improvement may range from 2 to 4 points on the RMDQ.^27,29,30^ The within-group RMDQ score change at 3 months was 3.0 points for the control group and 3.7 points for the intervention group. Although the between-group difference was smaller than the clinically relevant difference, the addition of the selfBACK system to usual care may potentially enable the achievement of a clinically meaningful within-group change. This hypothesis is supported by the substantially larger percentage of participants achieving a score improvement of 4 points or higher on the RMDQ in the intervention group compared with the control group.

### Strengths and Limitations

This trial has several strengths. First, it includes a patient-centered intervention, well-balanced baseline characteristics between groups, an intervention that was delivered according to protocol, a smaller loss to follow-up than anticipated, blinded analysis, and small but consistent between-group differences for the primary outcome and several disparate secondary outcomes that were sustained at 9 months. Second, the participants were recruited from diverse primary care settings, and there were few restrictions on participant characteristics and no upper limits on age, thereby enhancing the generalizability of the findings. Further research is needed to identify the active components of the intervention and the potential moderators, such as digital health literacy. Process evaluation,^45^ including interviews with participants and clinicians, may provide valuable insights into how to refine the selfBACK app to increase its effectiveness.

The trial also has some limitations. First, the participants were not blinded. However, participants in the intervention group did not receive additional attention from the researchers beyond the app installation and initial instructions. Nevertheless, this situation may have introduced a performance bias that overestimated the effect of the selfBACK system. Second, health care use was not monitored during the follow-up. A possible synergistic effect between self-management support and usual care cannot be excluded. Third, the step-detecting wristband worn by participants in the intervention group may have introduced an additional benefit that is independent of using the selfBACK app. Fourth, the per-protocol analyses could be biased if participants who engaged with the app during the follow-up period had a different prognosis from those who had little app usage. Further research is required to determine the cost-effectiveness and long-term benefits (beyond 9 months) of the selfBACK system.

## Conclusions

Among adults with LBP who sought care in a primary care or an outpatient spine clinic, those who received the AI-based selfBACK system as an adjunct to usual care had less LBP-related disability at 3 months compared with those who received usual care alone. This difference was sustained at 9 months. However, the improvement in pain-related disability was small and of uncertain clinical significance. Process evaluation may provide insights into refining the selfBACK app to increase its effectiveness.

## Notes

Supplement 1. **eAppendix.** Description of the Intervention **eTable 1.** Means (SD) and Adjusted Between-Group Differences for Primary Outcome at 3 and 9 Months for All Sensitivity Analyses **eTable 2.** Proportion of Participants Improved and Relative Risk Comparing Groups at 3 and 9 Months **eTable 3.** Odds Ratio for Secondary Binary Outcomes Comparing Groups at 3 and 9 Months **eTable 4.** Mean (SD) and Between-Group Differences for Exploratory Outcomes at 3 and 9 Months **eTable 5.** Odds Ratio for Exploratory Binary Outcomes Comparing Groups at 3 and 9 Months

Supplement 2.
Statistical Analysis Plan

Supplement 3.
Data Sharing Statement
