# Supplementary material for: Effectiveness of App-Delivered, Tailored Self-management Support for Adults With Lower Back Pain–Related Disability: A selfBACK Randomized Clinical Trial
Source: JAMA Intern Med. 2021 Aug 2;181(10):1–10. doi: 10.1001/jamainternmed.2021.4097 (PMC8329791; doi:10.1001/jamainternmed.2021.4097)
Supplement: Supplement 1. — eAppendix. Description of the Intervention eTable 1. Means (SD) and Adjusted Between-Group Differences for Primary Outcome at 3 and 9 Months for All Sensitivity Analyses eTable 2. Proportion of Participants Improved and Relative Risk Comparing Groups at 3 and 9 Months eTable 3. Odds Ratio for Secondary Binary Outcomes Comparing Groups at 3 and 9 Months eTable 4. Mean (SD) and Between-Group Differences for Exploratory Outcomes at 3 and 9 Months eTable 5. Odds Ratio for Exploratory Binary Outcomes Comparing Groups at 3 and 9 Months [file jamainternmed-e214097-s001.pdf]

Supplementary Online Content

Sandal LF, Bach K, Øverås CK, et al. Effectiveness of app-delivered, tailored self-management support for adults with lower back pain–related disability: a SELFBACK randomized clinical trial. *JAMA Intern Med.* Published online August 2, 2021. doi:10.1001/jamainternmed.2021.4097

eAppendix. Description of the Intervention

eTable 1. Means (SD) and Adjusted Between-Group Differences for Primary Outcome at 3 and 9 Months for All Sensitivity Analyses

eTable 2. Proportion of Participants Improved and Relative Risk Comparing Groups at 3 and 9 Months

eTable 3. Odds Ratio for Secondary Binary Outcomes Comparing Groups at 3 and 9 Months

eTable 4. Mean (SD) and Between-Group Differences for Exploratory Outcomes at 3 and 9 Months

eTable 5. Odds Ratio for Exploratory Binary Outcomes Comparing Groups at 3 and 9 Months

This supplementary material has been provided by the authors to give readers additional information about their work.

© 2021 Sandal LF et al. *JAMA Internal Medicine.*

eAppendix. Description of the Intervention

The SELFBACK decision support system

The following is a condensed description of the SELFBACK system. A more detailed description can be found in[1, 2]:

- Mork PJ, Bach K, SELFBACK Consortium. A Decision Support System to Enhance Self-Management of Low Back Pain: Protocol for the SELFBACK Project. *JMIR Res Protoc*. Jul 20 2018;7(7):e167. doi:10.2196/resprot.9379.
- Bach K, Szczepanski T, Aamodt A, Gundersen OE, Mork PJ. Case representation and similarity assessment in the SELFBACK decision support system. Paper presented at: Case-Based Reasoning Research and Development: 24th International Conference, ICCBR 2016; October 31 - November 2, 2016; Atlanta, GA, USA.

SELFBACK is an evidence-based decision support system that supports self-management of nonspecific low back pain. In specific, SELFBACK provides the user with evidence-based advice on physical activity level, strength/ flexibility exercises, and educational content. The self-management advice is delivered via a smartphone app and individually tailored to the user’s personal goals, personal characteristics, symptom progression and functional level. The SELFBACK system uses the case-based reasoning (CBR) methodology to capture and reuse knowledge from successful previous cases to suggest the most suitable self-management plan for a current user. Figure 1 illustrates the architecture of the SELFBACK system and the process for producing and tailoring the weekly self-management plans (steps 1-5).

In the current trial, patients with low back pain were referred to the research project from their primary care clinician (general practitioner, physiotherapist, chiropractor) or an outpatient spine clinic. The patient was screened for eligibility by a research assistant and if eligible, invited to the trial and sent a link to an online web-based questionnaire (step 1). The questionnaire information was used to create a user profile (step 2), initiate the first CBR cycle (i.e., matching of the current case with the most similar and successful previous case in the SELFBACK case-base), and produce the first weekly self-management plan. The resulting self-management plan is pushed to the mobile phone (step 3) and accessed by the user (step 4). On a weekly basis, the users answered a set of tailoring questions in the app (eg, pain intensity, self-efficacy level, fear-avoidance level, barriers to self-management etc.). In addition, physical activity was tracked by a step detecting wristband (Mi Band 3, Xiaomi) connected to the SELFBACK app. The self-reported data and the objective physical activity data for the past week was then fed back to the CBR system (step 5) where the refined and enhanced user profile was matched with the most similar and successful case in the case-base to create and tailor the next weekly self-management plan.

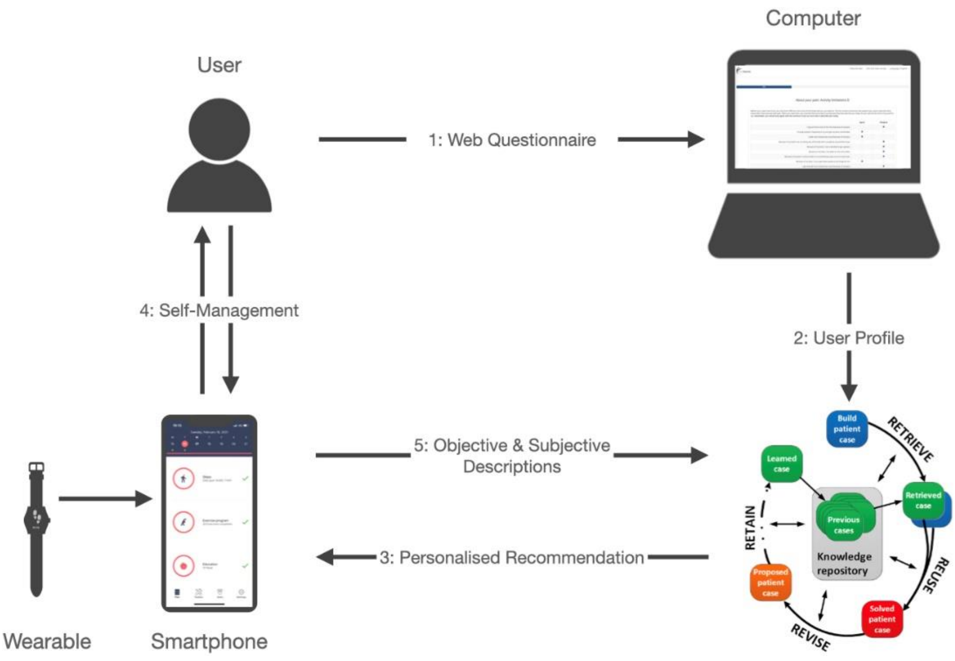

Figure 1. Illustration of the overall architecture of the SELFBACK system and how the modules link together to produce and tailor the weekly self-management plans (for more details, see Mork et al, 2018[1])

© 2021 Sandal LF et al. *JAMA Internal Medicine.*

The SELFBACK app

The following is a condensed description of the SELFBACK app. A more detailed description can be found in[3]:

- Sandal LF, Stochkendahl MJ, Svendsen MJ, et al. An App-Delivered Self-Management Program for People With Low Back Pain: Protocol for the selfBACK Randomized Controlled Trial. JMIR Res Protoc. Dec 3 2019;8(12):e14720. doi:10.2196/14720.

The SELFBACK app presents the self-management advice to the user.

When opening the app, the user is taken to the plan screen (Figure 2), which displays an overview of the daily self-management activities that can be performed by the users.

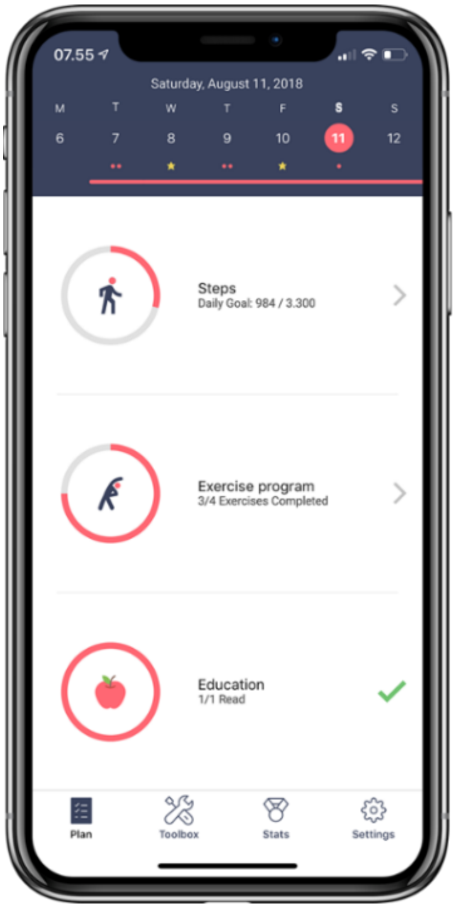

Figure 2. Screenshot of the plan screen from the SELFBACK app.

The top bar shows a timeline for the current week. Users can scroll flip through the days and review the activities completed on previous days and the recommended activities for the coming days. By selecting a previous date, the user can see a previous plan.

The current day is marked by the red circle.
The small red dots below the dates indicate number of activities completed. If all three components are completed within one day, a yellow star is displayed. The red bar below the dates indicate the duration of the current self-management plan (in this case, a new plan was created on Tuesday 7th August).

The three main components (ie. steps/physical activity, exercise, and education) are represented by separate ribbons and icons. The grey/red circle surrounding the icon shows the completion status for the activities. In this case, 994 steps of the goal of 3,300 step has been completed.

The ribbon at the bottom of the screen is a menu to navigate through different parts of the app and content.

The toolbox includes a library of educational messages read and exercises performed, tools (ie. goal setting, mindfulness audio), and educational items, such as 'about low back pain', etc.

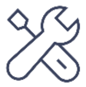

Overall statistics for all three components (eg. total number of steps, total number of educational items read etc.)

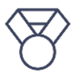

Settings where the users can adjust the app interaction (eg. turn notifications on/off).

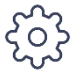

© 2021 Sandal LF et al. *JAMA Internal Medicine.*

From the plan screen, each of the three content ribbons can be clicked to enter a new subscreen.

Exercise

In the exercise screen an overview of exercises suggested in the current self-management plan is displayed (Figure 3).

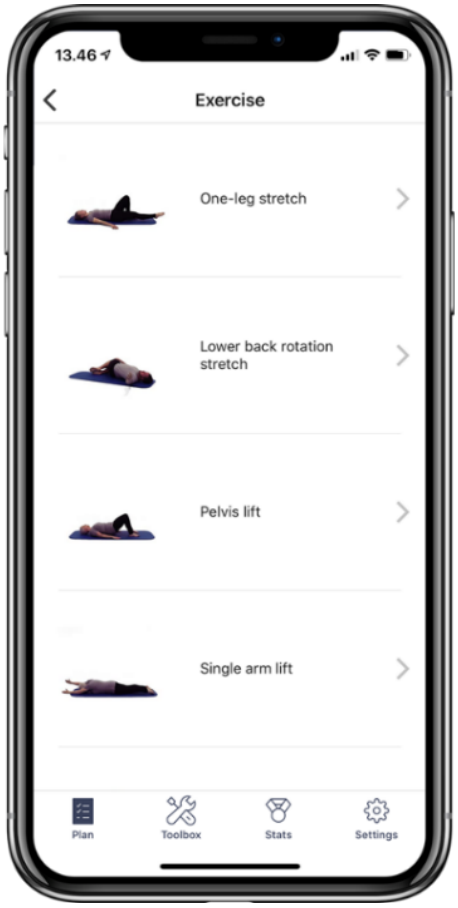

Figure 3. Screenshot of the exercise screen from the SELFBACK app.

All exercises are displayed with a small picture and a descriptive title.

By clicking the individual exercise, the user is shown a new screen including a video of the exercise as it should be performed and a detailed description of the exercise performance.

When a user has performed the exercise, they are taken to a reporting screen where they can enter the number of performed repetitions and sets.

Once an exercise is completed, it is “greyed out” in the overview (however, it can always be accessed at a later stage).

The user can choose to skip an exercise or replace an exercise with another exercise. If the user uses this option, s/he is asked to report the reason by choosing between the following response options: “too hard”, “not hard enough”, or ”pain during exercise”.

© 2021 Sandal LF et al. *JAMA Internal Medicine.*

Educational messages
When clicking the education ribbon in the plan screen, users are taken to new screen with an educational message showing a short informative title and a short message with a length of approximately 140 characters (Figure 4).

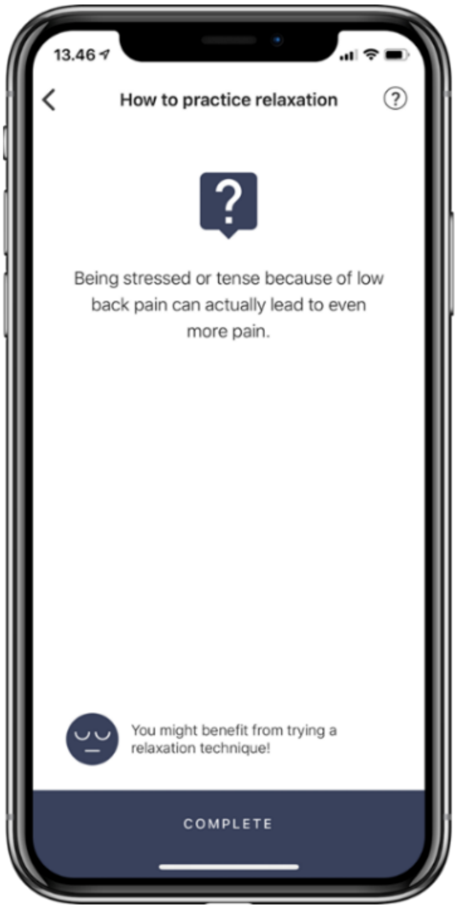

Figure 4. Screenshot of an educational screen from the SELFBACK app.

The short message may be displayed as a quiz, with response options “yes” or “no”. After the user indicates the response, s/he is taken to a new screen showing the correct answer and an explanation.

Some of the short messages are supplemented by a “read more” option, in which a longer message (≥500 characters) would appear below the short message.

The short messages can also be accompanied by links to tools within the app or longer reads placed in the general information section in the tools section.

The educational content is tailored to the individual user according to the answers on the baseline and follow-up questionnaires and weekly tailoring sessions.

© 2021 Sandal LF et al. *JAMA Internal Medicine.*

Physical activity
When clicking the physical activity ribbon in the plan screen, users are taken to a new screen providing statistics related to their level of physical activity (Figure 5).

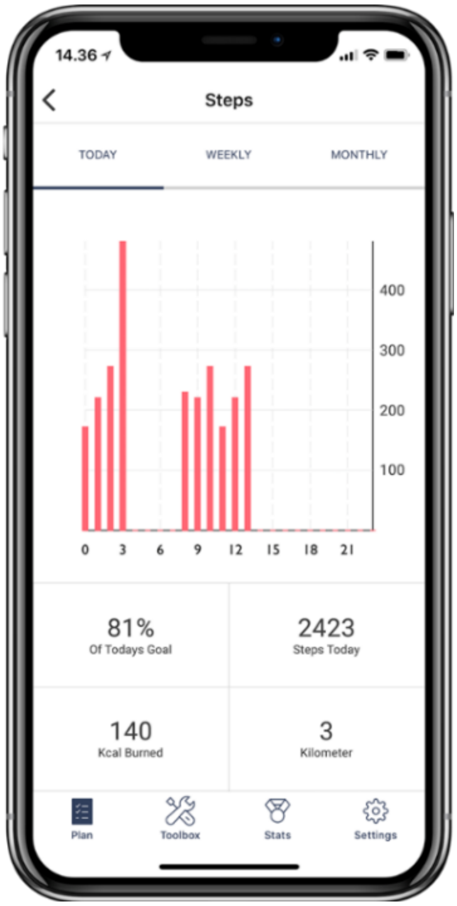

Figure 5. Screenshot of the physical activity screen from the SELFBACK app.

The number of steps is displayed, and the user can choose between a daily, weekly, or monthly overview.
The bottom part of the screen shows different statistics for the chosen timeframe.

The statistics include:

Percentage completion of the set goal for the day, average for the week or month.

Number of total steps for the day, week, or month.

An estimation of energy expenditure.

An estimation of the distance walked in kilometers for the day, week, or month.

© 2021 Sandal LF et al. *JAMA Internal Medicine.*

The content of the intervention

The development of the intervention and its related content followed a systematic six step process of Intervention Mapping outlined by Bartholomew et al.[4]. Through this process a logic model of the problem was outlined, followed by a formulation of program outcomes and objectives into a logic model of change to inform the program design and program production. The process is informed by literature and is evidence-based in its approach.

The content of the intervention was based on current treatment guidelines for low back pain, evidence-based treatment components and underpinned by theories for behavior change and engagement in digital health interventions.

The **exercise** content consisted of a bank of 70 exercises organized in 6 targets: 1) flexibility exercises, 2) pain relieving exercises in addition to strength exercises for 3) back extensors, 4) gluteal and hip muscles, 5) abdominal muscles, and 6) core muscles.

It is important to note that the individual user could adjust their time available for exercise in the tailoring session and consequently, the exercise dose performed would differ in both content, volume and intensity across the intervention group.

The **educational** content was organized in 14 overarching main themes with up to nine subthemes nested within them. Examples of main themes were "general information about low back pain", "how to overcome barriers for self-management of low back pain", "how to set SMART goals" and "low back pain and other medical conditions".

The **physical activity** screen showed continuous feedback on the number of steps and information on the completion level of the set goal.

The daily goal for number of steps was suggested by the SELFBACK system and thereafter adjusted by the individual user in the weekly tailoring session. The range for the step goal could range between 3,000 and 10,000 steps/day. The suggested daily step goal was calculated as a mean between the past week's set goal and the actual performance in the past week. If the suggested step goal was lower than 6,000 steps/day, the user could adjust this within a ±10% range. If the suggested step goal was between 6,000 and 8,000 steps/day, the users could adjust this within a ±15% range. If the suggested step goal was higher than 8,000 steps/day, the users could adjust this within a ±20% range.

© 2021 Sandal LF et al. *JAMA Internal Medicine.*

REFERENCES

1. Mork PJ, Bach K, selfBACK Consortium. A Decision Support System to Enhance Self-Management of Low Back Pain: Protocol for the selfBACK Project. *JMIR Res Protoc*. Jul 20 2018;7(7):e167. doi:10.2196/resprot.9379

2. Bach K, Szczepanski T, Aamodt A, Gundersen OE, Mork PJ. Case Representation and Similarity Assessment in the SELFBACK Decision Support System. *Case-Based Reasoning Research and Development, Iccbr 2016*. 2016;9969:32-46. doi:10.1007/978-3-319-47096-2_3

3. Sandal LF, Stochkendahl MJ, Svendsen MJ, et al. An App-Delivered Self-Management Program for People With Low Back Pain: Protocol for the selfBACK Randomized Controlled Trial. *JMIR Res Protoc*. Dec 3 2019;8(12):e14720. doi:10.2196/14720

4. Bartholomew Eldredge LK, Markham CM, Ruiter RAC, Kok G, Parcel GS, Fernández ME. *Planning Health Promotion Programs : An Intervention Mapping Approach*. 4th ed. John Wiley & Sons, Incorporated; 2016.

© 2021 Sandal LF et al. *JAMA Internal Medicine.*

eTable 1. Means (SD) and Adjusted Between-Group Differences for Primary Outcome at 3 and 9 Months for All Sensitivity Analyses

	Mean (SD)[a]		
	**Usual Care (n = 229)**	**SELFBACK[b] (n = 232)**	**Between-Group Differences. Adjusted[c] Mean (95% CI)**
**Roland-Morris Disability Questionnaire**			
**Sensitivity analyses**			
Multiple imputation analyses[d], (n = 461)			
Baseline	10.4 (4.4)		
3 months	7.4 (5.4)	6.6 (4.7)	-0.73 (-1.45 to -0.01)
9 months	6.7 (5.6)	5.9 (5.3)	-0.78 (-1.54 to -0.03)
Complete case analyses, (n = 290)			
Baseline	9.9 (4.2)		
3 months	7.1 (5.3)	6.4 (4.6)	-0.64 (-1.47 to 0.18)
9 months	6.7 (5.4)	5.7 (5.1)	-0.97 (-1.80 to -0.14)
Per protocol analyses[e], (n = 410)			
Baseline	10.4 (4.4)		
3 months	7.4 (5.4)	6.9 (4.8)	-0.61 (-1.36 to 0.14)
9 months	6.8 (5.6)	6.2 (5.0)	-0.75 (-1.54 to 0.04)

Abbreviations: SD, Standard Deviation; CI, Confidence Interval

[a] Marginal means from a crude linear mixed model and SDs from raw data among persons with information at the specific time points

[b] App-delivered self-management support in addition to usual care

[c] Adjusted for stratification variables (country and clinician), education ((<10, 10-12, >12 years), pain duration (<1, 1-4, 5-12, >12 weeks), average pain intensity past week at baseline (continuous, range 0-10), sex (male, female), age (years)

[d] Roland Morris Disability Questionnaire scores were imputed using a multivariate normal model with 20 imputations

[e] Adherence to the protocol was defined as creating at least 6 weekly self-management plans during the first 12 weeks post randomization for the intervention group

© 2021 Sandal LF et al. *JAMA Internal Medicine.*

eTable 2. Proportion of Participants Improved and Relative Risk Comparing Groups at 3 and 9 Months

	Usual care			SELFBACK[a]			
	No. Improved/ No. Total	Percentage Improved	Adjusted[b] Odds Ratio (95% CI)	No. Improved/ No. Total	Percentage Improved	Adjusted[b] Odds Ratio (95% CI)	Between-Group Differences. Adjusted[b] Odds Ratio[c] (95% CI)
**Improvement ≥2 points on RMDQ**							
Baseline	0/229	0	N/A	0/232	N/A	N/A	
3 months	114/190	60.0	0.98 (0.70 to 1.37)	143/209	68.4	1.41 (0.91 to 2.20)	1.44 (0.94 to 2.22)
9 months	115/182	63.0	1.13 (0.80 to 1.60)	112/170	65.0	1.25 (0.80 to 1.97)	1.11 (0.71 to 1.73)

Abbreviations: CI, Confidence Interval; RMDQ, Roland-Morris Disability Questionnaire; N/A, Not Applicable

[a] App-delivered self-management support in addition to usual care

[b] Adjusted for stratification variables (country and clinician), education (<10, 10-12, >12 years), pain duration (<1, 1-4, 5-12, >12 weeks), average pain intensity past week at baseline (continuous, range 0-10), sex (male, female), age (years)

[c] Usual care was used as reference group

© 2021 Sandal LF et al. *JAMA Internal Medicine.*

eTable 3. Odds Ratio for Secondary Binary Outcomes Comparing Groups at 3 and 9 Months

	Usual care		SELFBACK[a]		
	**No. Improved/ No. Total**	**Adjusted[b] Odds Ratio[c] (95% CI)**	**No. Improved/ No. Total**	**Adjusted[b] Odds Ratio[c] (95% CI)**	**Between-Group Differences. Adjusted[b] Odds Ratio[d] (95% CI)**
**Pain Self-Efficacy Questionnaire, >40**					
Baseline, both groups	302/460	1.00 (reference)	-	-	
3 months	133/188	1.39 (1.00 to 1.94)	173/205	2.82 (1.94 to 4.10)	2.02 (1.27 to 3.23)
9 months	140/181	1.88 (1.32 to 2.67)	143/169	2.53 (1.81 to 3.53)	1.35 (0.85 to 2.14)
**Fear-Avoidance Belief Questionnaire, >50 perc. (>10)**					
Baseline, both groups	221/460	1.00 (reference)	-	-	
3 months	69/188	0.63 (0.46 to 0.85)	79/205	0.73 (0.54 to 0.98)	1.16 (0.79 to 1.72)
9 months	61/181	0.56 (0.40 to 0.77)	47/169	0.46 (0.34 to 0.63)	0.82 (0.54 to 1.26)
**Average Pain Intensity Past Week, >5**					
Baseline, both groups	185/461	1.00 (reference)	-	-	
3 months	53/189	0.57 (0.42 to 0.78)	34/205	0.31 (0.21 to 0.45)	0.53 (0.34 to 0.84)
9 months	50/181	0.55 (0.40 to 0.77)	29/169	0.33 (0.23 to 0.48)	0.60 (0.38 to 0.95)
**Worst Pain Intensity Past Week, >5**					
Baseline, both groups	338/461	1.00 (reference)	-	-	
3 months	94/189	0.35 (0.26 to 0.48)	72/205	0.16 (0.12 to 0.23)	0.47 (0.31 to 0.70)
9 months	85/181	0.32 (0.22 to 0.44)	57/169	0.16 (0.11 to 0.23)	0.50 (0.32 to 0.76)
**Brief Illness Perception Questionnaire, >75 perc. (>52)**					
Baseline, both groups	100/461	1.00 (reference)	-	-	
3 months	36/187	0.79 (0.54 to 1.16)	21/205	0.37 (0.23 to 0.60)	0.47 (0.27 to 0.84)
9 months	26/181	0.50 (0.32 to 0.79)	16/169	0.39 (0.23 to 0.66)	0.77 (0.40 to 1.50)
**Saltin-Grimby Physical Activity Level, moderate/vigorous**					
Baseline, both groups	338/461	1.00 (reference)	-	-	
3 months	94/189	0.99 (0.75 to 1.29)	72/205	0.85 (0.65 to 1.10)	0.86 (0.60 to 1.23)
9 months	85/181	0.95 (0.70 to 1.31)	57/169	1.12 (0.83 to 1.50)	1.17 (0.79 to 1.75)
**Global Perceived Effect, improved[e]**					
Baseline, both groups	0/461		-	-	
3 months	99/187	1.44 (1.04 to 1.98)	149/205	3.42 (2.15 to 5.44)	2.38 (1.53 to 3.69)
9 months	91/181	1.22 (0.85 to 1.77)	129/168	4.17 (2.57 to 6.77)	3.41 (2.14 to 5.42)

Abbreviations: CI, Confidence Interval

[a] App-delivered self-management support in addition to usual care

[b] Adjusted for stratification variables (country and clinician), education (<10, 10-12, >12 years), pain duration (<1, 1-4, 5-12, >12 weeks), average pain intensity past week at baseline (continuous, range 0-10), sex (male, female), age (years)

[c] Baseline for both groups as reference group

[d] Usual care as reference group

[e] Usual care at 6 weeks as reference group

© 2021 Sandal LF et al. *JAMA Internal Medicine.*

eTable 4. Mean (SD) and Between-Group Differences for Exploratory Outcomes at 3 and 9 Months

	**Mean (SD)[a]**		
	**Usual Care (n = 229)**	**SELFBACK[b] (n = 232)**	**Between-Group Differences, Adjusted[c] Mean (95% CI)**
**Work ability (0-10)**			
Baseline	6.7 (2.0)		
3 months	7.1 (2.1)	7.4 (1.8)	0.24 (-0.13 to 0.61)
9 months	7.3 (2.0)	7.5 (1.9)	0.23 (-0.16 to 0.62)
**Perceived Stress Scale (0-40)**			
Baseline	14.9 (6.8)		
3 months	14.8 (7.2)	13.9 (7.1)	-0.78 (-1.74 to .018)
9 months	13.8 (7.1)	12.3 (7.1)	-1.31 (-2.31 to -0.31)
**Patient Health Questionnaire-8 (0-24)**			
Baseline	6.4 (4.3)		
3 months	6.2 (4.6)	5.8 (4.5)	-0.43 (-1.05 to 0.18)
9 months	5.8 (4.9)	5.1 (4.3)	-0.71 (-1.35 to -0.07)

Abbreviations: SD, Standard Deviation; CI, Confidence Interval
[a] Marginal means from a crude linear mixed model and SDs from raw data among persons with information at the specific time points
[b] App-delivered self-management support in addition to usual care
[c] Adjusted for stratification variables (country and clinician), education (<10, 10-12, >12 years), pain duration (<1, 1-4, 5-12, >12 weeks), average pain intensity past week at baseline (continuous, range 0-10), sex (male, female), age (years)

© 2021 Sandal LF et al. *JAMA Internal Medicine.*

eTable 5. Odds Ratio for Exploratory Binary Outcomes Comparing Groups at 3 and 9 Months

	Usual care		SELFBACK[a]		
	**No. Improved/ No. Total**	**Adjusted[b] Odds Ratio[c] (95% CI)**	**No. Improved/ No. Total**	**Adjusted[b] Odds Ratio[c] (95% CI)**	**Between-Group Differences. Adjusted[b] Odds Ratio[d] (95% CI)**
**Pain Medication, ≥1 day/week**					
Baseline, both groups[c]	367/461	1.00 (reference)	-	-	
3 months	159/189	1.49 (1.03 to 2.15)	186/205	2.65 (1.67 to 4.20)	1.78 (1.00 to 3.14)
9 months	159/181	2.17 (1.41 to 3.33)	150/169	2.03 (1.27 to 3.23)	0.93 (0.50 to 1.73)
**Work Ability, high (>7)**					
Baseline, both groups[c]	132/345	1.00 (reference)	-	-	
3 months	75/145	1.79 (1.30 to 2.46)	83/149	2.21 (1.51 to 3.23)	1.24 (0.79 to 1.95)
9 months	77/137	1.90 (1.31 to 2.77)	77/122	2.91 (1.89 to 2.48)	1.53 (0.91 to 2.57)
**Sleep Problems, insomnia**					
Baseline, both groups[c]	326/461	1.00 (reference)	-	-	
3 months	124/187	0.79 (0.59 to 1.06)	127/205	0.65 (0.47 to 0.89)	0.82 (0.55 to 1.21)
9 months	111/181	0.65 (.048 to 0.89)	98/169	0.55 (0.39 to 0.76)	0.84 (0.56 to 1.26)
**Perceived Stress Scale, high (>27)**					
Baseline, both groups[c]	12/461	1.00 (reference)	-	-	
3 months	9/187	2.49 (1.22 to 5.05)	9/205	1.67 (0.69 to 4.02)	0.67 (0.26 to 1.72)
9 months	2/181	0.68 (0.24 to 1.94)	3/168	0.64 (0.13 to 3.19)	0.94 (0.16 to 5.58)
**Perceived Health Questionaire-8, moderate/severe (>15)**					
Baseline, both groups[c]	20/461	1.00 (reference)	-	-	
3 months	8/187	0.99 (0.54 to 1.81)	9/205	1.05 (0.48 to 2.31)	1.06 (0.42 to 2.67)
9 months	9/181	1.31 (0.65 to 2.66)	6/168	0.99 (0.48 to 2.07)	0.76 (0.30 to 1.93)
**Patient Acceptable Symptom State, acceptable[e]**					
Baseline, both groups	N/A		N/A		
3 months	87/187	1.21 (0.88 to 1.67)	124/205	2.09 (1.36 to 3.21)	1.72 (1.13 to 2.62)
9 months	98/181	1.64 (1.19 to 2.27)	109/168	2.47 (1.59 to 3.82)	1.50 (0.98 to 2.31)

Abbreviations: CI, Confidence Interval
[a] App-delivered self-management support in addition to usual care
[b] Adjusted for stratification variables (country and clinician), education (<10, 10-12, >12 years), pain duration (<1, 1-4, 5-12, >12 weeks), average pain intensity past week at baseline (continuous, range 0-10), sex (male, female), age (years)
[c] Baseline for both groups as reference group
[d] Usual care as reference group
[e] Usual care at 6 weeks as reference group

© 2021 Sandal LF et al. *JAMA Internal Medicine.*
