# Supplementary material for: Effectiveness of App-Delivered, Tailored Self-management Support for Adults With Lower Back Pain–Related Disability: A selfBACK Randomized Clinical Trial
Source: JAMA Intern Med. 2021 Aug 2;181(10):1–10. doi: 10.1001/jamainternmed.2021.4097 (PMC8329791; doi:10.1001/jamainternmed.2021.4097)
Supplement: Supplement 2. — Statistical Analysis Plan [file jamainternmed-e214097-s002.pdf]

Effectiveness of a tailored app-delivered self-management program for reducing pain-related disability in people with low back pain: statistical analyses plan (SAP) for the SELFBACK randomised controlled trial.

Section 1: Administrative Information

SAP Version 0.2 (March 17th, 2020)

This SAP is based on the protocol registered in ClinicalTrials.gov (NCT03798288) and the published protocol paper: Sandal et al. (2020). Effectiveness of an App-Delivered Self-Management Program for People with Low Back Pain - a Protocol for the selfBACK Randomised Controlled Trial, *JMIR Research Protocols*[1]. The structure and content of the SAP is adopted from the Guidelines for the Content of Statistical Analyses Plans in Clinical Trials[2].

Table 1. SAP revision history

Revision	Justification for revision	Version (date)

This SAP is developed as a collaborative effort between all partners in the SELFBACK consortium, as described in the registered trial protocol (ref).

Signatures

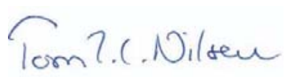

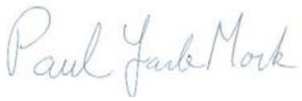

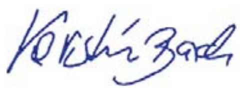

Senior Statistician | Project Coordinator | Project Manager

Section 2: Introduction

2.1 Background and rational

Low back pain (LBP) is a major contributor to years lived with pain and disability, and the societal costs related to healthcare, social benefits, sickness absence, and reduced work ability are high. Clinical guidelines recommend patient education, exercise therapy, multidisciplinary treatments and combined physical and psychological interventions to manage LBP, and self-management programmes that include these elements have been suggested as a promising option. Digital solutions, such as mobile applications (apps), have been suggested as platforms for supporting self-management of chronic conditions, but evidence of their quality and effectiveness is limited. The SELFBACK project aims to fill this knowledge gap by developing an evidence-based and data-driven decision support system (DSS) delivered via a smartphone app to facilitate, improve and reinforce self-management of non-specific LBP[3]. The DSS suggests self-management plans consisting of physical activity advice, patient education and recommendations for physical exercise tailored to the individual's specific health information. The effectiveness of the SELFBACK DSS will be evaluated in a randomized controlled trial (RCT).

2.2 Objectives

The objective is to evaluate the effectiveness of the SELFBACK DSS in addition to usual care versus usual care only in a RCT. Primary outcome is pain-related disability at three months measured by the Roland-Morris Disability Questionnaire (RMDQ). We hypothesise that participants randomised to using the SELFBACK app in addition to usual care will have at least two points difference in RMDQ at three months, compared to participants receiving usual care only.

The effect of the intervention on secondary outcomes, including quality of life, use of non-prescriptive medication, sleep problems, depressive symptoms, stress, functional ability and LBP intensity, will be assessed at three months. We will also evaluate the effect on these measures, as well as on RMDQ, at nine months.

Section 3: Study Methods

3.1 Trial design

The SELFBACK study is designed as an international multi-centre RCT where patients with non-specific LBP are randomised to two parallel groups (allocation ratio 1:1). The intervention group will be given access to the SELFBACK DSS, delivered via a smartphone app (SELFBACK app) in addition to usual care, whereas the control group will get usual care only. In addition, participants in the intervention group are given a wearable device (i.e., a step-detecting wristband) that interacts with the app. The target population are people seeking care for non-specific LBP from a primary care provider (general practitioner, physiotherapist, chiropractor) or an out-patient clinic (the Spine Centre of Southern Denmark); see also sections 5.2 and 5.3.

The SELFBACK system is a data-driven predictive DSS that uses case-based reasoning (CBR) methodology to capture and reuse participant cases in order to suggest the most suitable self-management plan for participants. Based on the step count and participant's self-reported data, the app provides individually tailored self-management plans, including

educational messages, physical activity advice and exercise recommendations matched to the participant's health status. The self-management plans are updated weekly based on the participant's achievements and symptom progress.

3.2 Randomization

Randomisation is performed as a block randomisation with permuted blocks of random size unknown to the research team and stratified by country (Denmark or Norway) and care provider (general practitioner, physiotherapist, chiropractor, or Spine Centre). Randomisation is performed by a web-based randomisation system (Web Case Report Form; WebCRF) developed and administered by Unit of Applied Clinical Research, Faculty of Medicine and Health Sciences, NTNU, Trondheim, Norway. This unit is not otherwise involved in the trial management or trial conduct.

3.3 Sample size

The sample size calculations are described in detail in the registered protocol. Briefly, the study aims to detect a two-point difference in pain related disability measured by the RMDQ at three months follow-up. Based on calculations and simulations, and assuming 30% drop-out, at least 350 participants (175 in each arm) will be included in the trial.

3.4 Framework

The SELFBACK study is designed as a superiority RCT assessing the effectiveness of the SELFBACK DSS in addition to usual care (intervention group) compared to usual care only (control group) for people with non-specific LBP.

3.5 Interim analyses and stopping guidance

As serious adverse events are unexpected, no interim analysis or a priori defined stopping rules are defined or implemented for this trial.

3.6 Timing of outcome assessment

The primary and secondary outcome variables will be assessed at baseline and at six weeks, three months, six months and nine months follow-up. This allows analyses of repeated measures on both primary and secondary outcomes, and thus increased statistical power compared to outcomes assessed at a single time-point. The main outcome of the study is pain related disability measured by RMDQ at three months follow-up.

3.7 Timing of final analyses

The analyses of the primary outcome will be conducted within six months after the last participant has completed the three months follow-up questionnaire. The inclusion of participants will last until December 2019, and thus the final analyses should be ready by end of September 2020. Analyses of secondary outcomes assessed at three months will be analysed subsequently to the primary outcome and conducted within the same time frame. The effect on outcomes assessed at nine months follow-up will be analysed after the final data collection, and the timing and focus for these analyses will be decided upon by the

partners involved in each sub-project. A data steering committee comprising one member from each participating partner will manage the use of the data.

Section 4: Statistical Principles

4.1 Confidence intervals and P-values

As recently recommended in the medical literature, we will not use a specific *P*-value threshold to decide upon statistical significance as this often leads to misinterpretation of results. For the same reasons, we will not adjust for multiple comparisons since this build upon a strict use of a certain *P*-value threshold. Instead, the precision of the estimated effects of the intervention will be assessed by a 95% confidence interval, and the effect will be described as a point estimate (mean difference or odds ratio) with accompanying confidence limits. Whenever *P*-values are reported, we will do so by presenting their actual value, and not reduce them to a binary inequality under or above a threshold value.

4.2 Adherence and protocol deviations

Since the intervention focuses on the participant's use and interaction with the SELFBACK DSS in the app, and since this interaction constitutes the basis for a self-management plan that is updated weekly, we will define adherence to the intervention as receiving an updated self-management plan for six of the first 12 weeks after enrolment. We will present numbers and the proportion within the intervention group adhering to the intervention and this will provide the basis for per protocol analyses. In sensitivity analyses we will also explore the influence of defining adherence differently to better capture cases who improve rapidly and do not need self-management support towards the end of the three months follow-up period, e.g. using four of the first six generated plans as criteria for adherence. Similarly, we will conduct sensitivity analyses where we aim at capturing more continuous use of the app throughout the follow up period; e.g. at least two plans generated during each four-week follow-up period.

Protocol deviations may occur due to technical failures at either the front end (i.e., app, smart phone or the wearable device) or the back end (server down-time) that causes users to be unable to interact with the DSS or not having a weekly plan generated. The number of protocol deviations, their reasons, and how many participants were affected will be summarised. Moreover, since some participants may answer the follow-up questionnaires at a somewhat later time point than the intended six weeks, three, six and nine months, we will report the variation in follow-up time for each period.

4.3 Analysis populations

The main effect of the intervention will be analysed according to the intention-to-treat principle using linear mixed models for continuous outcomes and generalized estimated equations (GEE) for binary outcomes, and the analyses will include all participants initially enrolled in the study and who answered the baseline questionnaire and were randomised. The web-based baseline questionnaire does not allow participants to proceed without filling in an answer, so there will be no missing data at this time point. A similar solution will be used for the follow-up questionnaires, but missing data will be generated if participants do not answer the follow-up questionnaire (i.e. due to withdrawal or loss to follow-up).

Any missing values throughout the follow-up period are inherently accounted for in the mixed model approach, but multiple imputation methods and complete case analysis will be applied in sensitivity analyses (see chapter 6.2 and 6.3 below for further details). A complete case will be defined as a participant who has answered both the baseline and the three-month questionnaire. We will also conduct per protocol analyses only including participants from the intervention arm who are defined as adhering to the intervention (i.e., having an updated self-management plan created during six of the 12 first weeks, see chapter 4.2 for more details).

Section 5: Trial Population

5.1 Screening data

The trial does not aim to collect any screening data to describe the representativeness of the sample.

5.2 Eligibility

Detailed inclusion and exclusion criteria are described in the registered protocol. Briefly, the participant must be ≥18 years and seek care from primary health-care practice or a specialised outpatient hospital facility (Spine Centre, see details below) for non-specific LBP within the past 8 weeks. They must also report mild-to severe pain-related disability (≥6 on RMDQ) and own a smart phone with internet access.

5.3 Recruitment

The recruitment of participants is conducted in Trondheim, Norway and Odense, Denmark. In both Norway and Denmark, participants are recruited from general practice, physiotherapy and chiropractic clinics. In Denmark, participants are also recruited from the Spine Centre of Southern Denmark. The recruitment started in March 2019 and will continue until December 2019. A total of 350 participants are to be recruited to the RCT; 75% (n=262) in Denmark and 25% in Norway (n=88). Further details on recruitment are given in the registered protocol.

5.4 Withdrawal and follow-up

Each participant is informed that they can withdraw from the study at any time, and that they then have the right to have any personal, health and questionnaire data deleted. If a participant withdraws during the follow-up period, but do not require already collected data to be deleted, the data will be used in the analyses until the time point for withdrawal. For analyses of the primary and secondary outcomes at three months, loss to follow-up is defined as not answering the three-month questionnaire. Loss-to-follow-up will be assessed for each outcome variable separately. For secondary outcomes measured at later follow-up points, loss to follow-up is defined according to the time-point when the last information is collected for that particular outcome. Number of participants providing information at each follow-up time point will be visualised in the CONSORT flow-chart, and this also displays the number who withdrew or were lost to follow-up between each follow-up time-point.

5.5. Baseline patient characteristics

Eligible participants fill in a baseline web questionnaire after verbally consenting to take part in the study. Baseline characteristics that are collected include: age, sex, height, weight, housing (live alone or with family), ethnicity, education, employment, work characteristics, physical activity, sleep problems, mental health, stress, quality of life, and various pain-related factors (e.g., localisation, duration, intensity, coping, disability and limitations, perceptions and beliefs). Depending on the nature of the variables, we will summarise this information in a baseline table showing mean values with standard deviation or numbers and percentages within the two trial arms (intervention and control). We will not conduct any statistical tests of baseline differences, as this violates the assumptions for the randomisation procedure.

Section 6: Analyses

6.1 Outcome definitions

All outcome variables described below are assessed at baseline, six weeks, three, six and nine months. The primary follow-up time point is three months, both for the primary and secondary outcome variables described below. In later analyses we will also assess more long-term effects using nine months as the follow-up time point. For each follow-up period, all previous measures will inform the repeated measures analyses (e.g. baseline and six-week data will be included when analysing effects at three months, and all previous time points will be included in repeated measures analyses of more long-term effects at nine months).

Primary outcome variable

The primary outcome is pain-related disability assessed at three months, measured by the RMDQ[4]. The questionnaire includes 24 items asking participants to indicate if they experience functional impairments by answering “yes” or “no” to a series of descriptions of functional abilities. The RMDQ score ranges from 0 to 24, where a higher score indicates higher levels of disability due to LBP. The main analyses will be based on the raw scores, and we will estimate mean group differences in RMDQ at three months using a linear mixed model for repeated measures. We will also construct a binary variable representing a clinically meaningful change in RMDQ of two points or more during the three months follow-up period that will be analysed using a logistic GEE analyses for repeated measures to estimate odds ratios between the groups. Since the size of a clinically meaningful change in RMDQ has been debated, we will also construct a binary variable representing a larger cut-off (e.g. 4 points change). For more details on these analyses, see chapter 6.2 below.

Secondary outcome variables

- *LBP intensity* within the past week will be assessed by asking “Please indicate your average/worst low back pain level during the last week“, using an 11-point numerical rating scale (NRS) ranging from “0 (zero)” to “10”[5]. We will compare mean group differences in LBP intensity using linear mixed models. We will also construct a binary variable to indicate moderate/severe pain (>5 points) that will be analysed using a logistic GEE analyses.

- *Pain medication* is informed by the question “How many days during the last week have you taken non-prescription pain medication for low back pain?” with four response options ranging from “never” to “daily”. The variable will be analysed both as an ordinal variable of the original scale and a binary variable classified as Never vs ≥1 time using logistic GEE analyses.
- *The Fear-Avoidance Belief Questionnaire* (FABQ) assesses participant’s beliefs about how physical activity and work affect their LBP[6]. The FABQ is a 5-item questionnaire, where the participants score their beliefs about their LBP on an ordinal scale ranging from “zero [completely disagree]” to “six [completely agree]”. The four latter questions will be summed (range 0-24) to represent fear avoidance beliefs about physical activity and analysed as a continuous variable to compare mean group differences using linear mixed models. We will also classify people as having high or low fear for physical activity to examine possible differences in a binary variable using logistic GEE analyses. The classification cut-off will be obtained from the distribution of the variable (e.g. median value).
- *The Pain Self-Efficacy Questionnaire* (PSEQ) assesses the participant’s level of confidence in carrying out specific activities despite their pain[7]. The PSEQ is a 10-item questionnaire scored on an ordinal scale ranging from “zero [completely disagree]” to “six [completely agree]”. A total score is calculated by summing the scores for each of the 10 items, yielding a maximum total score of 60, where higher scores reflect stronger self-efficacy beliefs. We will compare mean group differences in PSEQ using linear mixed models. We will also construct a binary variable to indicate low (<20) and high (<40) self-efficacy that will be analysed using logistic GEE .
- *Activity Limitation* due to LBP is measured by “Has low back trouble caused you to reduce your activity during the last 12 months? with response options “yes” and “no” to each of the domains work or leisure. The two variables will be analysed using logistic GEE to estimate odds ratios between the groups. A variable combining information from the two domains (i.e. activity limitations in both work and leisure) will also be constructed and analysed as a binary variable using logistic GEE.
- *Work ability* is measured by a single-item on current work ability rated on an 11-point NRS scale ranging from “zero [completely unable to work]” to “10 [work ability at its best]”[8]. We will compare mean difference in work ability using linear mixed models. We will also classify people into a binary variable representing high (>7 points) vs low work ability and analyse this using logistic GEE.
- *Self-reported physical activity* is evaluated by the Modernised Saltin-Grimby Physical Activity Level Scale, where participants indicate their amount of time per week performing leisure activities with four levels of intensity ranging from sedentary to vigorous physically active[9]. The resulting four categories will be analysed as an ordinal variable in logistic GEE, as well as collapsed into a binary variable indicating no/light activity vs moderate/vigorous activity.
- *Function* is evaluated by the Patient Specific Functional Scale (PSFS) where participants are asked to rate up to two self-selected activities they are unable to do or are having difficulties performing[10]. The ability to carry out the activity/activities is rated from “zero [unable to perform]” to “10 [fully able to perform]”. We will compare mean difference in function using linear mixed models, and also analyse a binary variable representing a clinically important improvement (≥3 points) in function for each of the two items using logistic GEE.
- *Sleep problems* is assessed by four items including problems with falling asleep, waking up repeatedly, waking up too early, and feeling sleepy during the day[11].

Response options for each item are “seldom or never”, “sometimes” or “several times a week”. The information retrieved from these four items approximates the information necessary to diagnose insomnia according to the DSM-V criteria. The resulting binary variable (insomnia vs no insomnia) will be analysed using logistic GEE to estimate odds ratios for insomnia between the groups.

- *Stress* is evaluated with the Perceived Stress Scale (PSS), a 10-item questionnaire asking about frequency of thoughts and feelings related to perceived stress[12]. Participants indicate their frequency of experiencing stress-related issues on a 5-point Likert scale, ranging from “0 [never]” to “4 [very often]”. Positive score items are reversed and then all items are summed to a score ranging from 0 to 40. The resulting sum score will be analysed as a continuous variable to estimate mean differences in stress using linear mixed models. Cut-offs at 14 and 27 to indicate low, moderate and high stress will be used to construct a binary variable for analyses using logistic GEE.
- *Health-related quality of life* is evaluated with the EuroQoL 5-dimension (EQ-5D) questionnaire[13]. A 5-point Likert scale ranging from “1 [no problems]” to “5 [complete inability]” is used to assess the health-related quality of life within each of the five dimensions (i.e., mobility, self-care, activities, pain/discomfort and anxiety/depression). The five dimensions will be analysed separately as ordinal variables using logistic GEE. We will also construct an overall index based on value sets from Denmark and Norway that combines all items and then estimate the mean difference between groups using linear mixed models.
- *General health* is assessed on a 100 point vertical scale where 0 indicates the worst health you can imagine and 100 the best imaginable health[13]. The variable will be analysed as a continuous variable estimating the mean difference between groups using linear mixed models.
- *The Brief Illness Perception Questionnaire* (BIPQ) evaluates the participants’ illness perception in an 8-item questionnaire[14]. Items are scored on an ordinal scale ranging from “0 [no problems]” to “10 [worst severity]”. Adding the separate score values creates a summary score with a higher score indicating more threatening view of the pain. The summed score will be analysed as a continuous variable to compare mean group differences, and we will also construct a binary variable with cut-offs indicated from the distribution of the variable (e.g. percentiles) since no clinical cut offs are suggested in the literature.
- *The Patient Health Questionnaire-8* (PHQ-8) is an 8-item questionnaire used to evaluate the participants’ depressive symptoms[15]. Items are reported on a 4-point Likert scale scoring frequency of experiencing symptoms of depression. The nine items will be summed and analysed both as a continuous variable using linear mixed models and as a categorical variable using cut-offs of 5, 10, 15 and 20 to classify people into none, mild, moderate, moderately severe, and severe categories using logistic GEE for ordinal and binary (±15) variables.
- *Patient Acceptable Symptom State* asks “Considering your low back pain, do you consider your current state satisfactory?” with response options yes or no[16]. This will be analysed as a binary variable using logistic GEE.
- *Patient’s Global Perceived Effect* is a single item question where participants are asked to rate improvement or deterioration of their LBP compared to before the intervention with seven response options ranging from -5 [markedly worse] to 5 [markedly better][17]. The variable will be analysed as an ordinal variable using logistic GEE, and also collapsed into a binary variable indicating improved vs not improved.
- *Current pain duration* is measured by the question “What is the length of time you have had low back pain during this episode?” with four response options ranging from

"less than 1 week" to "more than 12 weeks". Although this information is collected at follow-up, the variable will not be included as a secondary outcome due to possible confusion about pain episode and the time frame for the episode.

- *Long term pain duration* is measured by "What is the total length of time that you have had low back trouble during the last 12 months?" with five response options ranging from "0 days" to "every day". Similar to the above, although this information is collected at follow-up the variable will not be included as a secondary outcome due to possible overlapping time frame with baseline and follow-up time points.

6.2 Analyses methods

The primary analysis will estimate mean difference and 95% confidence interval (CI) in RMDQ score at three months follow-up between the intervention and control group (i.e., SELFBACK in addition to usual care versus usual care only). The analyses will be conducted according to the intention-to-treat principle using a linear mixed model for repeated measures. This model includes all available data for all participants at each time point (i.e. baseline, six weeks, and three months). The distribution of the RMDQ score will be assessed, and the variable may be transformed (e.g. log transformation) to better fit with the assumptions for the regression analyses. In the regression model, individual participants will be specified as a random effect, accounting for the within subject covariance structure. The effect of group and time will be specified as fixed effects using a joint variable of intervention and time. Here, baseline levels are pooled over the two study groups assuming that any baseline differences are due to chance; this also controls for any baseline differences in the outcome variable. The between group difference at tree months will be adjusted for the two variables used for stratification in the randomisation (i.e., country and care provider). Further adjustment for baseline levels of potentially important prognostic factors, such as age, sex, socioeconomic status, pain duration, and pain intensity will also be conducted. We will also use GEE analyses to estimate an odds ratio (with 95% CI) for a two-point change in RMDQ between the groups taking into account the repeated observations. This analysis will be adjusted for the same factors as those included in the linear mixed model.

To reduce the risk of biased interpretation of results for the primary outcome we will use the following procedure: Two interpretations will be drafted based on the results from the main analyses, with interventions groups arbitrarily labelled as A and B. One interpretation assumes that A is the SELFBACK DSS in addition to usual care and B is usual care, the other interpretation assumes that A is the usual care and B is the selfBACK DSS in addition to usual care. After agreeing on both interpretations, the randomisation code is broken, and the correct interpretation will be used.

In addition to the intention to treat analyses, we will conduct per protocol analyses using information on adherence to the trial as described in chapter 4.2 above.

All secondary outcomes will be analysed using a similar approach; for continuous (or approximately continuous) we will use linear mixed models to estimate mean differences between groups, and for ordinal or binary variables we will use logistic GEE analyses to estimate odds ratios. Pre-specified cut-offs for ordinal and binary variables are described in 6.1 above. For analyses of mean differences, the distribution of each outcome variable will be assessed to inform possible transformation or initiate alternative analytical procedures (e.g. non-parametric analyses). The precision of all estimated effects will be assessed by a 95% CI.

Possible modifiers of the effect of intervention on the primary outcome will be assessed in supplementary analyses stratified by sex, age groups, socioeconomic status and different levels of LBP severity etc., and accompanied by tests of statistical interaction to

assess departure from additive effects (i.e., including a product term of group and modifier in the regression model).

6.3 Missing data

Any missing values are inherently accounted for in the mixed model approach, but multiple imputation methods and complete case analysis will be applied in sensitivity analyses. Multiple imputation will include factors that predict missingness of a specific factor, as well as all factors that are included in the main model (outcome, intervention, or adjustment variables). The number of imputed datasets will be guided by the number of missing observations for each variable, but we aim at using a minimum of 10 imputed datasets for each variable.

6.4 Additional analyses

Additional analyses include per protocol analyses, analyses of secondary outcomes, analyses stratified by possible effect modifiers, analyses using multiple imputation of missing data and complete case analyses. These analyses are described above in chapter 6.1-6.3.

6.5 Harms

As stated above, no harms are expected, and thus we do not plan any specific analyses for this. If any study related harms should occur, these will be described and reported.

6.6 Statistical software

All analyses related to the primary outcome will be conducted using Stata, whereas analyses of secondary outcomes may be done using other statistical software packages, such as SPSS or R.

References

1 Sandal, L. F. *et al.* An app-delivered self-management program for people with low back pain: Protocol for the selfBACK randomized controlled trial. *JMIR Research Protocols* **8**, e14720, (2019).

2 Gamble, C. *et al.* Guidelines for the content of statistical analysis plans in clinical trials. *JAMA* **318**, 2337-2343, (2017).

3 Mork, P. J. & Bach, K. A decision support system to enhance self-management of low back pain: Protocol for the selfBACK project. *JMIR Research Protocol* **7**, e167, (2018).

4 Roland, M. & Fairbank, J. The Roland-Morris Disability Questionnaire and the Oswestry Disability Questionnaire. *Spine* **25**, 3115-3124, (2000).

5 Hawker, G. A. *et al.* Measures of adult pain: Visual Analog Scale for Pain (VAS Pain), Numeric Rating Scale for Pain (NRS Pain), McGill Pain Questionnaire (MPQ), Short-Form McGill Pain Questionnaire (SF-MPQ), Chronic Pain Grade Scale (CPGS), Short Form-36 Bodily Pain Scale (SF-36 BPS), and Measure of Intermittent and Constant Osteoarthritis Pain (ICOAP). *Arthritis Care Res* **63 Suppl 11**, S240-252, (2011).

6 Waddell, G. *et al.* A Fear-Avoidance Beliefs Questionnaire (FABQ) and the role of fear-avoidance beliefs in chronic low back pain and disability. *Pain* **52**, 157-168, (1993).

7 Nicholas, M. K. The pain self-efficacy questionnaire: Taking pain into account. *Eur J Pain* **11**, 153-163, (2007).

8 Ahlstrom, L. *et al.* The work ability index and single-item question: associations with sick leave, symptoms, and health - a prospective study of women on long-term sick leave. *Scand J Work Environ Health* **36**, 404-412, (2010).

9 Grimby, G. *et al.* The "Saltin-Grimby Physical Activity Level Scale" and its application to health research. *Scand J Med Sci Sports* **25 Suppl 4**, 119-125, (2015).

10 Horn, K. K. *et al.* The patient-specific functional scale: Psychometrics, clinimetrics, and application as a clinical outcome measure. *J Orthop Sports Phys Ther* **42**, 30-42, (2012).

11 Engstrøm, M. *et al.* The reliability of a new sleep screening questionnaire for large population-based studies: The third Nord-Trøndelag Health Study. *Open Sleep Journal* **4**, 14-19, (2015).

12 Cohen, S. *et al.* A global measure of perceived stress. *J Health Soc Behav* **24**, 385-396, (1983).

13 Brooks, R. EuroQol: the current state of play. *Health policy (Amsterdam, Netherlands)* **37**, 53-72, (1996).

14 Hallegraeff, J. M. *et al.* Measurement of acute nonspecific low back pain perception in primary care physical therapy: reliability and validity of the brief illness perception questionnaire. *BMC Musculoskelet Disord* **14**, 53, (2013).

15 Kroenke, K. *et al.* The PHQ-9: Validity of a brief depression severity measure. *J Gen Intern Med* **16**, 606-613, (2001).

16 Conrozier, T. *et al.* Getting better or getting well? The patient acceptable symptom state (PASS) better predicts patient's satisfaction than the decrease of pain, in knee osteoarthritis subjects treated with viscosupplementation. *Cartilage* **9**, 370-377, (2018).

17 Kamper, S. J. *et al.* Global Perceived Effect scales provided reliable assessments of health transition in people with musculoskeletal disorders, but ratings are strongly influenced by current status. *J Clin Epidemiol* **63**, 760-766.e761, (2010).
