# Supplementary material for: Effectiveness of App-Delivered, Tailored Self-management Support for Adults With Lower Back Pain–Related Disability: A selfBACK Randomized Clinical Trial
Source: JAMA Intern Med. 2021 Aug 2;181(10):1–10. doi: 10.1001/jamainternmed.2021.4097 (PMC8329791; doi:10.1001/jamainternmed.2021.4097)
Supplement: Supplement 3. — Data Sharing Statement [file jamainternmed-e214097-s003.pdf]

Data Sharing Statement

Sandal. Effectiveness of App-Delivered, Tailored Self-management Support for Adults With Lower Back Pain-Related Disability. *JAMA Intern Med*. Published August 02, 2021.
doi:10.1001/jamainternmed.2021.4097

Data
Data available: Yes
Data types: Other (please specify)
How to access data: Data is kept for 5 years (reidentifiable data) and thereafter the data will be anonymised and stored up to 30 years.
When available: beginning date: June 1, 2021

Supporting Documents
Document types: None

Additional Information
Who can access the data: Researchers whose proposed use of the data has been approved by the data steering group
Types of analyses: Any relevant research purpose
Mechanisms of data availability: After approval by the data steering group. Contact to Paul Jarle Mork (paul.mork@ntnu.no) or Karen Søgaard (ksogaard@health.sdu.dk)
